# Mediterranean Diet and SARS-COV-2 Infection: Is There Any Association? A Proof-of-Concept Study

**DOI:** 10.3390/nu13051721

**Published:** 2021-05-19

**Authors:** Valentina Ponzo, Marianna Pellegrini, Chiara D’Eusebio, Fabio Bioletto, Ilaria Goitre, Silvio Buscemi, Simone Frea, Ezio Ghigo, Simona Bo

**Affiliations:** 1 Department of Medical Sciences, University of Torino, 10126 Torino, Italy; valentina.ponzo@unito.it (V.P.); mariannapellegrini87@gmail.com (M.P.); chiara.deusi@gmail.com (C.D.); fabio.bioletto@unito.it (F.B.); ilaria.goitre@libero.it (I.G.); ezio.ghigo@unito.it (E.G.); 2 Unit of Clinical Nutrition, AOU Policlinico “P. Giaccone”, 90127 Palermo, Italy; silvio.buscemi@unipa.it; 3 Dipartimento di Promozione della Salute, Materno-Infantile, Medicina Interna e Specialistica di Eccellenza (PROMISE), University of Palermo, 90127 Palermo, Italy; 4 Cardiology Unit, Città della Salute e della Scienza Hospital, University of Torino, 10126 Torino, Italy; frea.simone@gmail.com

**Keywords:** Mediterranean diet, SARS-COV-2 infection, healthcare professionals, dietary habits

## Abstract

The aim of this observational study was investigating the possible correlation between adherence to the Mediterranean diet (MeD) and SARS-COV-2 infection rates and severity among healthcare professionals (HCPs). An online self-administrated questionnaire (evaluating both MeD adherence and dietary habits) was filled out by HCPs working in Piedmont (Northern Italy) from 15 January to 28 February 2021. Out of the 1206 questionnaires collected, 900 were considered reliable and analyzed. Individuals who reported the SARS-COV-2 infection (*n* = 148) showed a significantly lower MeD score, with a lower adherence in fruit, vegetables, cereals, and olive oil consumption. In a logistic regression model, the risk of infection was inversely associated with the MeD score (OR = 0.88; 95% CI 0.81–0.97) and the consumption of cereals (OR = 0.64; 0.45–0.90). Asymptomatic individuals with SARS-COV-2 infection reported a lower intake of saturated fats than symptomatic; individuals requiring hospitalization were significantly older and reported worse dietary habits than both asymptomatic and symptomatic individuals. After combining all symptomatic individuals together, age (OR = 1.05; 1.01–1.09) and saturated fats intake (OR = 1.09; 1.01–1.17) were associated with the infection severity. HCPs who reported a SARS-COV-2 infection showed a significantly lower MeD score and cereal consumption. The infection severity was directly associated with higher age and saturated fat intake.

## 1. Introduction

Since March 2020, the world community is facing one of the biggest pandemics in its history and the first of the globalized world [1]. Almost no country has been spared from the spread of SARS-COV-2, but large differences exist in the prevalence rate among and within states [2]. In Italy, a north–south gradient in incidence and mortality is traceable, with northern regions showing the highest infection rates (6749.68 per 100,000 inhabitants), while the southern regions display the lowest rates (2188.10 per 100,000) [3,4]. Establishing the reasons for these differences could be very challenging, and many factors, such as different genetic backgrounds, lifestyles, higher age, comorbidities, air pollution, smoking habits, population density, and healthcare system organizations, may play a role [5,6,7]. Differences in dietary habits were hypothesized early on as critical factors in the variable susceptibility and severity of the infection owing to the role of nutrition in the human immune defense against virus attacks [8,9]. The intake of specific foods, such as fermented foods, ameliorated the outcomes of patients, while others, such as sugar products, adversely impacted the recovery from the virus infection [9,10]. Deficiencies in micronutrients (selenium, vitamin C, vitamin D) have been associated both with SARS-COV-2 infection and adverse outcomes in observational or ecological studies [11,12]. A few clinical trials reported favorable outcomes in patients with SARS-COV-2 infections after vitamin C [13] and D supplementation [14,15,16,17,18,19], with less severe disease and better survival. Finally, in Italy, the pandemic itself, owing to the psychological consequences and limitations imposed by the lockdown, determined worse lifestyle patterns with more frequent unhealthy food choices, as well as a reduction both in the adherence to the Mediterranean diet (MeD) and in the level of exercise [19,20,21,22,23,24].

The MeD is a dietary pattern rich in plant foods (fruits, vegetables, cereals, legumes, tree nuts, seeds, and olives): olive oil is the principal source of added fat, along with high to moderate intake of fish and seafood (≥2 servings/week), moderate weekly consumption of eggs (2–4/week), poultry, and dairy products (2 servings/week), low consumption of red meat (<2 servings/week), processed meat (≤1 serving/week), and sweets (≤2 servings/week), and a moderate intake of alcohol (mainly wine during meals) [25]. This combination of dietary habits results in high intakes of nutritional components with anti-inflammatory, antioxidant, and immunomodulatory effects, including dietary fiber, unsaturated fats, omega-3 polyunsaturated fatty acids (PUFAs), vitamins, minerals, and various healthy phytochemicals, such as polyphenols and flavonoids [26,27]. Previous meta-analyses suggested an inverse association between MeD adherence and the risk of all-cause mortality [28,29], cardiovascular diseases, cancer, and neurodegenerative and dysmetabolic diseases [30,31,32]. Moreover, the MeD was found to decrease inflammation, with a reduction in C-reactive protein (CRP) and proinflammatory cytokines [33,34,35], as well as a reduction in the risk of respiratory infections [36] and sepsis [37]. The trend of MeD adherence grows from the North to the South in Italy, while the SARS-COV-2 incidence rate rises in the opposite direction [3,4]. It has been hypothesized that the MeD may represent a potential approach to address conditions associated with SARS-COV-2 infection and severity, such as diabetes, cardiovascular diseases, and obesity [38,39,40]. To the best of our knowledge, no study has specifically addressed the association between the adherence to the MeD and the incidence of the SARS-COV-2 infection.

Healthcare professionals (HCPs), due to the nature of their work, are at increased risk of SARS-COV-2 infection. In Italy, 126,394 cases occurred among healthcare personnel, corresponding to 3.8% of total infections [3]. Incidence of SARS-COV-2 infection among the HCPs was reported to be around 10% [41,42].

The purpose of the present proof-of-concept observational study was therefore to investigate the possible correlations between adherence to the MeD and SARS-COV-2 infection rates and severity among HCPs. 

## 2. Materials and Methods

This was an observational retrospective study. Health professionals from the hospitals and the local health facilities in the Piedmont region were invited to participate by e-mail. The study was conducted from the 15 January 2021 to the 28 February 2021. Inclusion criteria were as follows: health professionals still active at work and in contact with patients, age 20 to 65 years, the ability to receive e-mails, and the ability to fill in an online questionnaire. Exclusion criteria were as follows: age <20 and >65 years, retirees, and people who were no longer active at work or who had no contact with patients. Data were collected through Google Forms, via e-mail.

### 2.1. Ethical Issues

The study protocol was approved by the Bioethics Committee of the University of Torino (number 492927, 4 December 2020). All the procedures were in line with the declaration of Helsinki. The research was conducted online, according to the recommendations of the Association of Internet Researchers [43]. All data were anonymous. Participants were informed about the aims of the study and were reassured about the fact that all information would be kept confidential and used for research purposes only; they were required to accept the data sharing and privacy policy before starting to fill in the questionnaire. Google Forms has privacy standards, including data protection.

### 2.2. Questionnaires

A self-administered questionnaire collecting clinical and dietary data was created by using Google Forms. The questionnaire included both the Medi-lite questionnaire [44] and a previously validated medium-length food-frequency questionnaire [45], plus items relative to sociodemographic and clinical characteristics. 

The questionnaire was first sent electronically to a small number of volunteers (*n* = 20) in order to check the feasibility, comprehensibility, and the time required for compilation. Only minor corrections relative to the wording of some sentences were made. The link to the finalized survey was sent by e-mail to the institutional e-mail addresses of HCPs from hospitals and local health facilities of different areas of the Piedmont region. The participants completed it autonomously and were strongly requested to be truthful and to answer carefully in order to ensure the quality of the survey. All the items were mandatory, and the respondents had the possibility to change their answers.

The following data were collected: age, actual working activity, gender, weight, height, education level, living alone or with friends/family, presence of chronic diseases (diabetes mellitus, arterial hypertension, cardiovascular, pulmonary, autoimmune diseases, others), and chronic use of drugs. Those who had received any type of degree (either a bachelor’s degree or a master’s degree) were considered as graduates. Furthermore, participants were asked about having ever contracted the SARS-COV-2 infection. They could choose one of the following answers: never, asymptomatic with a positive swab, home-managed infection, infection requiring hospital admission in a non-intensive care unit, or infection requiring the admission to an intensive care unit. 

The validated Medi-lite adherence score [46] assesses the consumption of nine food categories: the daily frequency of consumption of fruit, vegetables, cereals, meat and meat products, dairy products, alcohol, and olive oil, and the weekly frequency of consumption of legumes, and fish. The highest categories of consumption of the foods that are typical of the MeD (fruit, vegetables, cereals, legumes, and fish) received a score of 2 points, the middle categories 1 point, and the lowest categories 0 points. On the other hand, the highest categories of consumption of the food that are not typical of the MeD (meat and meat products and dairy products) received a score of 0 points, the middle categories 1 point, and the lowest categories 2 points. The daily consumption of 1–2 alcohol units (1 unit = 12 g alcohol) received 2 points, the daily consumption of <1 alcohol unit received 1 point, a daily alcohol consumption >2 units received 0 points. The regular consumption of olive oil received 2 points, the frequent consumption 1 point, and the occasional consumption 0 points [46]. The final score, i.e., the sum of the points, ranges from 0 (low adherence) to 18 (high adherence). 

The medium-length food-frequency questionnaire is a tool validated in the Italian population [45]. The questionnaire assessed the semi-quantitative habitual intake of different foods during the previous 12 months. In particular, the consumption of 36 different food items based on commonly eaten foods and portion sizes of the Italian population was collected. Participants indicated the usual consumption frequency of each food and the relative amount [45]. Photographs of food were used to help in the description of the portion size [47]. The food items were categorized as drinks (coffee, alcoholic, and soft drinks), milk and dairy products, meat–fish–eggs, cereals, vegetables–legumes–fruit, and fatty dressings and other (sweets, fried foods, and fast food) [45]. 

The intakes of energy and nutrients were calculated taking into account the portion size, the frequency of consumption, and the weighted mean energy and nutrient value of each food and beverage by using a Microsoft Excel software based on the most recent Council for Agricultural Research and Economics (CREA) Food Composition Database [48]. All questionnaires were analyzed by a registered dietitian, and a medical doctor (MD) with expertise in nutrition carefully checked for errors. Both of them were blinded to the answers relative to the SARS-COV-2 infection.

### 2.3. Validity of Self-Reported Energy Intake

The plausibility of self-reported energy intakes was assessed in accordance with literature [49]. Predicted energy requirements were calculated using the Schofield equations, in line with the Italian guidelines [50]. The measure of physical activity was not available; all participants were assumed to have a sedentary level of activity. This assumption is reasonable considering the significant reduction in exercise described during the pandemic period in Italy owing to the limitations imposed by the lockdown [24] and the fact that our participants were HCPs, all actively engaged in the patient care.

Participants were classified as under-reporters, plausible reporters, or over-reporters on the basis of their ratio of reported energy intake to predicted energy requirements. In order to account for the normal variation in energy expenditure and measurement errors, the confidence limits were calculated in line with literature: the ratio for under- and over-reporters were <0.70 and >1.42, respectively [51].

### 2.4. Statistical Methods

Anonymous data were collected, exported, and stored in password protected computers. Results were expressed as mean ± standard deviation (SD) or percentage as appropriate.

The Student’s *t*-test, ANOVA, and Mann–Whitney and Kruskal–Wallis tests were used for normally distributed or skewed variables, respectively. Post hoc analyses with the Scheffé test were employed for testing differences among subgroups. The Chi-square test was used to test for categorical variables. 

The independent association between the incidence of SARS-COV-2 infection and the variables that were significantly different at univariate analyses was assessed by two logistic regression models, one including the MeD score and the other including its single significant components. Due to the low number of subjects requiring hospital admission, the independent association between the severity of SARS-COV-2 infection and the variables that were significantly different at univariate analyses was assessed after combining symptomatic individuals in a unique category; moreover, due to the low number of patients who were available for this analysis, a stepwise backward selection approach was adopted to exclude from the model all variables losing statistical significance at multivariate analysis, thus reducing potential concerns for overfitting effects. Results were reported as odds ratio (OR) and 95% confidence intervals (CI). A *p*-value < 0.05 was considered statistically significant.

## 3. Results

After removing duplicate submissions, we recruited 1206 participants. Out of them, 294 (24.4%) were under-reporters, and 12 (1.0%) were over-reporters, thus leaving 900 individuals for the final analyses (Table 1). They were highly educated middle-aged adults, on average healthy (more than three-quarters without chronic diseases), and with normal weight; about half of them were MDs. The adherence to the MeD was found to have a slightly left-skewed distribution (skewness: −0.41) with a mean score of 10.5 (median 11.0) and a range from 2 to 17 (Table 2). 

In this sample, 148 (16.4%) reported the SARS-COV-2 infection; in 4% (*n* = 36) of participants, there was an occasional finding of positivity to the swab to which HCPs are periodically submitted in Italy, while 11.4% reported the occurrence of a symptomatic infection, and 1% reported the need of hospital admission (Table 3). Individuals who reported the SARS-COV-2 infection (any severity) showed a significantly higher intake of saturated fats and proteins, and a lower consumption of carbohydrates and fiber. The MeD score was significantly lower in the former, with a lower adherence relatively to the consumption of fruit, vegetables, cereals, and olive oil. By comparing scores as categorical variables (0,1,2) the results were similar (Supplementary Table S1). In a logistic regression model, each 1-point increment in the MeD score was associated with a 12% reduction in the risk of SARS-COV-2 infection (Table 4, Model 1). In the model including the single MeD components (Table 4, Model 2), the consumption of cereals was inversely associated with the odds of infection. The results did not change significantly if considering the scores as categorical variables (Supplementary Table S2). 

Among individuals with SARS-COV-2 infection, asymptomatic individuals were significantly younger and reported a lower intake of saturated fats than the symptomatic ones (Table 3). The small number of HCPs who underwent hospitalization were significantly older and reported a lower health status and worse dietary habits than both asymptomatic and symptomatic individuals (Table 3 and Supplementary Table S3). Their MeD score was 6.6. After combining symptomatic individuals in a unique category (Supplementary Tables S4 and S5), the significant variables were considered for inclusion in a logistic regression model through a stepwise backward selection; those that finally retained a statistically significant association with infection severity were age (OR 1.05; 95% CI 1.01–1.09) and percentage of saturated fats (OR 1.09; 95% CI 1.01–1.17). This result did not vary when including, among the starting variables of the model, the MeD components as continuous or categorical variables. 

## 4. Discussion

A significantly lower MeD score was found in HCPs who reported a SARS-COV-2 infection, in particular a lower cereal consumption. Furthermore, the infection severity was directly associated with increasing age and saturated fat intake.

### 4.1. SARS-COV-2 Incidence

We found lower mean values of the MeD score than reported in other Italian studies employing the Medi-lite questionnaire [52,53,54]. Differences might be due to the demographic characteristics of our participants, composed of middle-aged active-working HCPs from northern Italy, since higher MeD scores have been reported in the elderly and in unemployed or low-occupational level individuals when compared to skilled workers [52,53]. Furthermore, a lower adherence to MeD was described in northern compared to southern Italian regions, both in adults and teenagers [55,56,57], even if an overall trend towards reduction in the MeD adherence was observed nationwide over the years, particularly among younger people [58,59,60].

The MeD score was significantly lower in our HCPs with a SARS-COV-2 infection and, among them, in those with symptomatic disease and more severe infection. These results suggest a potential protective effect of the MeD against the SARS-COV-2 infection, as proposed by several authors [38,39,40]. In a recent ecological study, MeD adherence was negatively associated with both SARS-COV-2 cases and related deaths [61]. However, the design of the study did not allow for establishing a direct relationship, and many confounding factors related to societal norms, cultural factors, the governmental response to the pandemic, and differences in healthcare systems across countries may have influenced the results [61]. 

To the best of our knowledge, our study is the first that specifically evaluated the association between MeD adherence and SARS-COV-2 infection. A few studies have assessed the link between a high MeD adherence and the generic risk of infections, reporting a lower risk for sepsis in adults [37], and better outcomes of children with recurring colds and frequent inflammatory complications [36]. 

The beneficial effects of the MeD are related to its high content of immunomodulating and anti-inflammatory substances, such as vitamins, PUFAs, polyphenols, and fibers. A beneficial role of vitamins against SARS-COV-2 infection has been hypothesized [62]. Antioxidant vitamins could reduce the generation of reactive oxygen (ROS) and reactive nitrogen species in response to the infection, which has been shown to be harmful to host cells [63]. PUFAs, in particular omega-3, exert anti-inflammatory effects as they are metabolic precursors of specialized pro-resolving lipid mediators, including α-linolenic acid, eicosapentaenoic acid, and docosahexaenoic acid, which could contribute to dampening the critical inflammatory period [64]. Polyphenols, whose intake was higher in the presence of a high MeD adherence [65], interact with transcription factors, including nuclear factor kappa-light-chain-enhancer of activated B cells (NF-kB) and nuclear factor erythroid 2-related factor 2 (Nrf-2), exerting anti-inflammatory and antioxidant effects, respectively [63].

Furthermore, the MeD has been reported to modulate the platelet-activating factor (PAF), a glyceryl-ether phospholipid (1-O-alkyl-2-acetyl-sn-glycero-3-phosphocholine) which is a mediator of inflammation and thrombosis [66] and might contribute to the thromboembolic complications that are common in severe SARS-COV-2 infections [40]. MeD was reported to reduce PAF both in healthy subjects and in patients with type 2 diabetes mellitus [67,68], and the healthy components of the MeD (cereals, legumes, vegetables, fish, and wine) favorably modulate the pro-inflammatory actions of PAF [69]. 

Finally, a plethora of epidemiological studies have demonstrated the benefits of increasing adherence to the MeD towards weight control and the risk of obesity and dysmetabolic diseases [70]. Excess of adiposity is a well-known risk factor for severe disease and mortality in patients with SARS-COV-2 infection, particularly in the elderly, owing to common underlying pathophysiological features [38,71]. Adhering to the MeD might therefore confer benefits towards the SARS-COV-2 infection by means of its favorable role on weight control.

Our results showed that the consumption of cereals was inversely associated with the risk of SARS-COV-2 infection. Unfortunately, the questionnaires we used did not allow us to discriminate the types of consumed cereals (whole or refined). Evidence from epidemiological data showed that whole cereals consumption is associated with reduced risks for developing chronic diseases, such as type 2 diabetes mellitus, cardiovascular diseases, obesity, and some types of cancer [72,73,74]. Moreover, improvements in insulin resistance, blood lipid levels, and markers of subclinical systemic inflammation have been associated with the intake of whole grains [75,76,77], with several of their components implicated in these beneficial effects, such as fiber, magnesium, betaine, resistant starch, and phytochemicals, including polyphenols and lignan [78,79]. On the other hand, the intake of refined cereals, despite being widely assumed to be associated with adverse health outcomes, failed to be linked to cardiometabolic diseases and all-cause mortality by several meta-analyses [80,81,82,83], while total grain consumption was associated with a reduced risk of chronic diseases and mortality [84,85], even if to a lesser extent than whole grains. Cereal grains seem to display an immunomodulatory activity by inducing the production of interleukin-10 (IL-10) from CD14+ monocytes in vitro [86,87]; polyphenols and β-glucans, major components of the soluble fraction of the whole grain fiber, have been suggested to stimulate immune responses [88,89], and, together with other types of fiber, might contribute to the modulation of the gut microbiota, a critical player in the development of the host immune system [90].

### 4.2. SARS-COV-2 Severity

We found a direct association between SARS-COV-2 infection severity and age, in line with the literature [91]. The elderly showed a longer diseases course, worse clinical patterns, poorer outcomes during hospitalization, and an increased risk of death [92,93,94]. The phenomenon known as inflammaging, the inflammatory state associated with aging as part of the decline in the effectiveness of the immune system termed immuno-senescence, might contribute to the infection severity [95].

The consumption of saturated fats (SFAs) was significantly higher among HCPs reporting a symptomatic SARS-COV-2 infection. Both clinical and preclinical studies reported an association between dietary SFA intake and inflammation markers and chronic systemic low-grade inflammation [96,97,98,99]. Low-grade chronic inflammation, a common condition in people with obesity and diabetes mellitus, has been proposed to facilitate the cytokine storm, which is linked to SARS-COV-2 infection severity [100,101]. The mechanisms by which SFAs trigger chronic low-grade inflammation are not completely understood. These compounds are able to activate Toll-like receptor-4 (TLR4) signaling [102], a member of the family of Toll-like receptors which, after recognizing exogenous pathogen-associated molecular components and various non-microbial damage-associated molecular patterns, promote the synthesis of pro-inflammatory cytokines and chemokines and induce the expression of co-stimulatory molecules, such as CD80 (cluster of differentiation) and CD86, on antigen-presenting dendritic cells, thus triggering adaptive immune responses [102]. Furthermore, SFAs might amplify inflammation through the increased synthesis of ceramides (bioactive sphingolipids mediating numerous cell-signaling events), which enhance the NF-κB pathway, thus increasing the expression of several pro-inflammatory genes [103,104]. Finally, SFAs might contribute to low-grade systemic inflammation by modulating the gut microbiota composition. High SFA diets have been implicated in reducing the gut microbiota richness, increasing the Firmicutes-to-Bacteroidetes ratio, and inducing gut dysbiosis [105,106]. This latter condition leads to an increased intestinal permeability with enhanced lipopolysaccharides (LPS) transport in the bloodstream, resulting in metabolic endotoxemia [107], which contributes to low-grade inflammation, insulin resistance, adipocyte hyperplasia, and the reduction of pancreatic beta-cells function [108,109].

### 4.3. Limitations

Our prevalence of under-reporters was in line with the previous literature [110,111]. The employed questionnaires did not allow us to properly estimate the micronutrient intakes of our participants; furthermore, we did not collect data about the intake of vitamin D or other supplements. This did not enable us to specifically test the association between infection rate and nutrients, such as vitamins and trace elements, that have been demonstrated to play key roles in supporting the immune system and helping in deal with coronavirus infections [112]. The enrolment of HCPs from a single region and the low percentage of male participants were further study limitations. Indeed, there is a gender bias in participating in the completion of food questionnaires [113,114]. Furthermore, in Italy, there is a predominance of women among healthcare workers (>70% in Piedmont) [115]; this finding is reassuring about the representativeness of our sample. This was a proof-of-concept study and, therefore, no explicit estimations of the sample size were made at the time of the study design. However, in a post hoc analysis, given the observed results and considering 0.05 as significance level, this study had a power of 99.7% for the detection of differences in the MeD score between subjects who reported the SARS-COV-2 infection and those who did not, and a power of 73.9% for the detection of differences in the MeD score between subjects requiring hospitalization and those reporting an infection of any other severity. 

## 5. Conclusions

Health care professionals reporting a SARS-COV-2 infection had a significantly lower MeD score, in particular, a lower than recommended consumption of cereals. Among those with the infection, higher age and increased saturated fat intake were correlated with a more severe disease. These results are worth confirming in a larger series of individuals with SARS-COV-2 infections of greater severity. The potential role of the specific MeD foods and/or their key components warrants further investigation as well, and clinical explorative trials on this topic are urgently needed. 

In conclusion, the MeD might represent a potential approach to address the SARS-COV-2 outbreak in order to prevent and/or improve disease-related outcomes.

## Figures and Tables

**Table 1:** Characteristics of the participants.

		SARS-COV-2		*p*
		Yes	No	
Number	900	148	752	
Age (years)	41.8 ± 13.1	43.3 ± 12.5	41.5 ± 13.2	0.13
Males (%)	27.7	27.7	27.7	0.99
Graduation (%)	90.1	89.9	90.2	0.91
Living alone (%)	14.4	10.1	15.3	0.10
MD (%)	50.7	53.4	50.1	0.47
No chronic diseases (%)	75.7	73.0	76.2	0.40
Weight (kg)	64.3 ± 12.3	65.6 ± 12.9	64.0 ± 12.2	0.16
Height (cm)	167.8 ± 8.4	167.9 ± 8.2	167.8 ± 8.4	0.82
BMI (kg/m^2^)	22.8 ± 3.7	23.2 ± 4.1	22.7 ± 3.6	0.10

MD: medical doctor; BMI: body mass index.

**Table 2:** Dietary habits of the participants.

		SARS-COV-2		*p*
		Yes	No	
Number	900	148	752	
Total energy (kcal)	1992.1 ± 468.9	1937.3 ± 464.8	2002.9 ± 469.2	0.12
Total carbohydrates (% kcal)	49.1 ± 7.8	47.7 ± 8.8	49.4 ± 7.5	0.018
Sugars (% kcal)	12.6 ± 5.4	12.2 ± 5.4	12.7 ± 5.3	0.35
Total fats (% kcal)	32.4 ± 6.7	33.1 ± 7.0	32.2 ± 6.6	0.15
Saturated fats (% kcal)	11.9 ± 6.0	13.1 ± 6.1	11.7 ± 6.0	0.015
Monounsaturated fats (% kcal)	15.9 ± 4.5	15.6 ± 4.7	15.9 ± 4.4	0.44
Polyunsaturated fats (% kcal)	4.5 ± 1.5	4.4 ± 1.6	4.6 ± 1.5	0.23
Proteins (% kcal)	16.4 ± 3.4	17.0 ± 3.4	16.3 ± 3.4	0.022
Fiber (g/day)	22.8 ± 7.4	20.6 ± 7.3	23.2 ± 7.3	<0.001
Alcohol (g/day)	6.0 ± 8.6	6.2 ± 8.4	5.9 ± 8.6	0.80 *
Mediterranean score				
Milk	0.60 ± 0.80	0.51 ± 0.72	0.61 ± 0.82	0.30 *
Meat	1.28 ± 0.80	1.21 ± 0.81	1.29 ± 0.80	0.24 *
Fruit	1.00 ± 0.88	0.76 ± 0.82	1.04 ± 0.89	<0.001 *
Vegetables	1.17 ± 0.87	0.92 ± 0.87	1.22 ± 0.86	<0.001 *
Legumes	1.09 ± 0.63	1.01 ± 0.61	1.11 ± 0.64	0.08 *
Cereals	1.82 ± 0.50	1.68 ± 0.67	1.85 ± 0.46	0.001 *
Fish	0.77 ± 0.54	0.76 ± 0.55	0.78 ± 0.54	0.68 *
Olive oil	1.71 ± 0.57	1.59 ± 0.66	1.74 ± 0.55	0.004 *
Alcohol	1.09 ± 0.39	1.09 ± 0.41	1.09 ± 0.39	0.83 *
Total score	10.5 ± 2.7	9.5 ± 2.8	10.7 ± 2.7	<0.001 *

MD = medical doctor; BMI = body mass index * Mann–Whitney test.

**Table 3:** Characteristics of the participants by the severity of the SARS-COV-2 infection.

	Asymptomatic	Symptomatic	Hospital Admission	*p*
Number	36	103	9	
Age (years)	37.2 ± 11.1	44.4 ± 12.5 *	54.8 ± 5.7 * §	<0.001
Males (%)	25.0	29.1	22.2	0.83
Graduation (%)	99.4	89.3	77.8	0.32
Living alone (%)	16.7	8.7	0.0	0.23
MD (%)	50.0	55.3	44.4	0.74
No chronic diseases (%)	77.8	74.8	33.3 * §	0.021
Weight (kg)	62.6 ± 11.4	66.2 ± 13.1	70.4 ± 15.0	0.19
Height (cm)	168.4 ± 7.5	167.8 ± 8.3	167.1 ± 9.3	0.89
BMI (kg/m^2^)	22.1 ± 3.7	23.4 ± 4.1	25.2 ± 4.8	0.08
Total energy (kcal)	1865.0 ± 327.4	1970.1 ± 503.8	1850.7 ± 467.9	0.43
Total carbohydrates (% kcal)	49.8 ± 7.1	47.9 ± 8.6	37.4 ± 11.0 * §	<0.001
Sugars (% kcal)	11.5 ± 4.4	12.2 ± 5.1	16.1 ± 10.0	0.07
Total fats (% kcal)	31.0 ± 5.5	33.1 ± 6.8	41.5 ± 9.1 * §	<0.001
Saturated fats (% kcal)	10.5 ± 5.4	13.3 ± 5.5 *	20.5 ± 8.4 * §	<0.001
Monounsaturated fats (% kcal)	15.9 ± 4.1	15.4 ± 4.8	16.8 ± 5.8	0.67
Polyunsaturated fats (% kcal)	4.6 ± 1.5	4.3 ± 1.6	4.3 ± 1.5	0.66
Proteins (% kcal)	17.1 ± 3.8	16.8 ± 3.3	18.3 ± 3.2	0.44
Fiber (g/day)	20.8 ± 6.4	21.0 ± 7.4	14.6 ± 6.6 §	0.036
Alcohol (g/day)	5.7 ± 5.8	6.1 ± 8.8	8.2 ± 12.9	0.45 **
Mediterranean score				
Milk	0.47 ± 0.77	0.53 ± 0.71	0.33 ± 0.71	0.52 **
Meat	1.25 ± 0.80	1.21 ± 0.82	1.00 ± 0.71	0.63 **
Fruit	0.89 ± 0.89	0.73 ± 0.79	0.56 ± 0.88	0.47 **
Vegetables	1.17 ± 0.91	0.87 ± 0.84	0.44 ± 0.88	0.05 **
Legumes	0.89 ± 0.62	1.09 ± 0.60	0.56 ± 0.53	0.018 **
Cereals	1.86 ± 0.49	1.68 ± 0.66	1.00 ± 1.00	0.004 **
Fish	0.75 ± 0.55	0.79 ± 0.55	0.44 ± 0.53	0.20 **
Olive oil	1.67 ± 0.59	1.60 ± 0.65	1.22 ± 0.97	0.40 **
Alcohol	1.19 ± 0.40	1.07 ± 0.40	1.00 ± 0.50	0.23 **
Total score	10.1 ± 3.1	9.6 ± 2.5	6.6 ± 3.1 * §	0.011 **

MD = medical doctor; BMI = body mass index * *p* < 0.05 vs. asymptomatic; § *p* < 0.05 vs. symptomatic (Scheffé test) ** Kruskal–Wallis test.

**Table 4:** Association between SARS-COV-2 infection and adherence to the MeD by logistic regression analyses.

**SARS-COV-2 Infection (*n* = 900)—Model 1**			
	**OR**	**95% CI**	***p***
Total carbohydrate (%kcal)	0.99	0.96–1.03	0.71
Saturated fats (%kcal)	1.00	0.96–1.04	0.99
Protein (%kcal)	1.02	0.97–1.09	0.42
Fiber (g/day)	0.98	0.95–1.02	0.31
MeD score	0.88	0.81–0.97	0.010
**SARS-COV-2 Infection (*n* = 900)—Model 2**			
	**OR**	**95% CI**	***p***
Total carbohydrate (%kcal)	1.01	0.97–1.05	0.70
Saturated fats (%kcal)	1.00	0.96–1.05	0.77
Protein (%kcal)	1.05	0.98–1.11	0.15
Fiber (g/day)	0.98	0.94–1.02	0.29
Fruit	0.85	0.64–1.13	0.27
Vegetables	0.86	0.63–1.19	0.37
Cereals	0.64	0.45–0.90	0.010
Olive oil	0.95	0.65–1.40	0.81
**Severity of the SARS-COV-2 Infection (*n* = 148) §**			
	**OR**	**95% CI**	***p***
Age (years)	1.05	1.01–1.09	0.006
Saturated fats (%kcal)	1.09	1.01–1.17	0.029

CI: confidence intervals; § *p* < 0.05 vs. symptomatic (Scheffé test). § Model obtained after stepwise backward selection of predictive variables.

## Data Availability

The data presented in this study are available on request from the corresponding author.

## Supplementary Materials

The following are available online at https://www.mdpi.com/article/10.3390/nu13051721/s1, Table S1: Mediterranean scores by SARS-COV-2 infection, Table S2: Association between SARS-COV-2 infection (yes vs no) and adherence to the MeD (categorical scores) by logistic regression analyses, Table S3: Mediterranean scores by SARS-COV-2 severity, Table S4: Characteristics of the participants by the severity of the SARS-COV-2 infection, after combining symptomatic individuals in a unique category, Table S5: Mediterranean scores by SARS-COV-2 severity, after combining symptomatic individuals in a unique category.
