# Supplementary material for: Mediterranean Diet and SARS-COV-2 Infection: Is There Any Association? A Proof-of-Concept Study
Source: Nutrients. 2021 May 19;13(5):1721. doi: 10.3390/nu13051721 (PMC8160854; doi:10.3390/nu13051721)
Supplement: Supplementary file 1 [file nutrients-13-01721-s001.zip › nutrients-1199218-supplementary.pdf]

Table 1 Supplementary. Mediterranean scores by SARS-COV-2 infection

		**SARS-COV-2**		P
	All	yes	No	
Number	900	148	752	
Milk				
0	60.6	62.8	60.1	
1	19.2	23.7	18.4	
2	20.2	13.5	21.5	0.052
Meat				
0	22.3	24.3	21.9	
1	27.7	30.4	27.1	
2	50.0	45.3	50.9	0.45
Fruit				
0	39.3	48.7	37.5	
1	21.8	27.0	20.7	
2	38.9	24.3	41.8	**<0.001**
Vegetables				
0	30.4	41.9	28.2	
1	22.2	24.3	21.8	
2	47.3	33.8	50.0	**<0.001**
Legumes				
0	15.9	18.2	15.4	
1	59.2	62.8	58.5	
2	24.9	18.9	26.1	0.17
Cereals				
0	5.2	11.5	4.0	
1	7.3	8.8	7.1	
2	87.4	79.7	89.0	**<0.001**
Fish				
0	28.8	30.4	28.5	
1	65.2	63.5	66.6	
2	6.0	6.1	6.0	0.89
Olive oil				
0	6.1	9.5	5.5	
1	16.2	21.6	15.2	
2	77.7	68.9	79.4	**0.017**
Alcohol				
0	3.7	4.1	3.6	
1	83.8	82.4	84.0	
2	12.6	13.5	12.4	0.89

Table 2 Supplementary. Association between SARS-COV-2 infection (yes *vs* no) and adherence to the MeD (categorical scores) by logistic regression analyses

	OR	95% CI	P
Total carbohydrate (%kcal)	1.00	0.97-1.04	0.84
Saturated fats (%kcal)	1.01	0.96-1.05	0.83
Protein (%kcal)	1.05	0.98-1.11	0.16
Fiber (g/day)	0.99	0.95-1.02	0.45
Fruit*			
0	1.00		
1	1.38	0.82-2.32	0.23
2	0.70	0.39-1.25	0.23
Vegetables*			
0	1.00		
1	0.83	0.47-1.49	0.54
2	0.67	0.35-1.28	0.23
Cereals*		0.45-0.90	
0	1.00		
1	0.38	0.15-0.93	**0.035**
2	0.33	0.16-0.69	**0.003**
Olive oil*			
0	1.00		
1	0.91	0.42-1.96	0.82
2	0.86	0.38-1.96	0.73

*Categorical variables: 0=0 points at the Medi-Lite adherence score (reference category); 1=1 point at the Medi-Lite adherence score; 2=2 points at the Medi-Lite adherence score

Table 3 Supplementary. Mediterranean scores by SARS-COV-2 severity

	**Asymptomatic**	**Symptomatic**	**Hospital admission**	**p**
Number	36	103	9	
Milk				
0	69.4	59.2	77.8	
1	13.9	28.2	11.1	
2	16.7	12.6	11.1	0.40
Meat				
0	22.2	25.2	22.2	
1	30.6	28.2	55.6	
2	47.2	46.6	22.2	0.51
Fruit				
0	44.4	48.5	66.7	
1	22.2	30.1	11.1	
2	33.3	21.4	22.2	0.43
Vegetables				
0	33.3	41.8	77.8	
1	16.7	29.1	0.0	
2	50.0	29.1	22.2	**0.024**
Legumes				
0	25.0	13.6	44.4	
1	61.1	64.1	55.6	
2	13.9	22.3	0.0	0.08
Cereals				
0	5.6	10.7	44.4	
1	2.8	10.7	11.1	
2	91.7	78.6	44.4	**0.008**
Fish				
0	30.6	28.2	55.6	
1	63.9	65.1	44.4	
2	5.6	6.8	0	0.51
Olive oil				
0	5.6	8.7	33.3	
1	22.2	22.3	11.1	
2	72.2	68.9	55.6	0.14
Alcohol				
0	0.0	4.9	11.1	
1	80.6	83.5	77.8	
2	19.4	11.7	11.1	0.40

Table 4 Supplementary. Characteristics of the participants by the severity of the SARS-COV-2 infection, after combining symptomatic individuals in a unique category.

	**Asymptomatic**	**Symptomatic**	**p**
Number	36	112	
Age (years)	37.2±11.1	45.2±12.4	**<0.001**
Males (%)	25.0	28.6	0.68
Graduation (%)	99.4	88.4	0.30
Living alone (%)	16.7	8.0	0.14
MD (%)	50.0	54.5	0.64
No chronic diseases (%)	77.8	71.4	0.46
Weight (kg)	62.6±11.4	66.5±13.3	0.12
Height (cm)	168.4±7.5	167.8±8.4	0.69
BMI (kg/m^2)	22.1±3.7	23.6±4.2	0.06
Total energy (kcal)	1865.0±327.4	1960.5±500.1	0.29
Total carbohydrates (% kcal)	49.8±7.1	47.0±9.2	0.10
Sugars (% kcal)	11.5±4.4	12.5±4.7	0.35
Total fats (% kcal)	31.0±5.5	33.7±7.4	**0.046**
Saturated fats (% kcal)	10.5±5.4	13.9±6.1	**0.004**
Monounsaturated fats (% kcal)	15.9±4.1	15.5±4.9	0.70
Polyunsaturated fats (% kcal)	4.6±1.5	4.3±1.6	0.37
Proteins (% kcal)	17.1±3.8	17.0±3.3	0.82
Fiber (g/day)	20.8±6.4	20.5±7.6	0.82
Mediterranean score			
Milk	0.47±0.77	0.52±0.71	0.53*
Meat	1.25±0.80	1.20±0.81	0.77*
Fruit	0.89±0.89	0.71±0.80	0.32*
Vegetables	1.17±0.91	0.84±0.84	0.05*
Legumes	0.89±0.62	1.04±0.61	0.18*
Cereals	1.86±0.49	1.63±0.71	**0.046***
Fish	0.75±0.55	0.76±0.56	0.94*
Olive oil	1.67±0.59	1.57±0.68	0.54*
Alcohol	1.19±0.40	1.06±0.41	0.10*
Total score	10.1±3.1	9.3±2.7	0.16*

MD=medical doctor; BMI=body mass index *Mann-Whitney test.

Table 5 Supplementary. Mediterranean scores by SARS-COV-2 severity, after combining symptomatic individuals in a unique category

	Asymptomatic	Symptomatic	p
Number	36	112	
Milk			
0	69.4	60.7	
1	13.9	26.8	
2	16.7	12.5	0.27
Meat			
0	22.2	25.0	
1	30.6	30.4	
2	47.2	44.6	0.94
Fruit			
0	44.4	50.0	
1	22.2	28.6	
2	33.3	21.4	0.34
Vegetables			
0	33.3	44.6	
1	16.7	26.8	
2	50.0	28.6	0.06
Legumes			
0	25.0	16.1	
1	61.1	63.4	
2	13.9	20.5	0.40
Cereals			
0	5.6	13.4	
1	2.8	10.7	
2	91.7	75.9	0.12
Fish			
0	30.6	30.4	
1	63.9	63.4	
2	5.6	6.3	0.99
Olive oil			
0	5.6	10.7	
1	22.2	21.4	
2	72.2	67.9	0.65
Alcohol			
0	0.0	5.4	
1	80.6	83.0	
2	19.4	11.6	0.20
